# Tissue ischemia microdialysis assessments following severe traumatic haemorrhagic shock: lactate/pyruvate ratio as a new resuscitation end point?

**DOI:** 10.1186/1471-2253-14-118

**Published:** 2014-12-15

**Authors:** Filip Burša, Leopold Pleva, Jan Máca, Peter Sklienka, Pavel Ševčík

**Affiliations:** Department of anesthesiology and intensive care medicine, University Hospital Ostrava, Faculty of Medicine Universitas Ostrava, 17. listopadu, 1790 Ostrava-Poruba, Czech Republic; Traumatology Centre, University Hospital Ostrava, Faculty of Medicine Universitas Ostrava, 17. listopadu, 1790 Ostrava-Poruba, Czech Republic

**Keywords:** Microdialysis, Shock, Lactate, Pyruvate, Haemoglobin, Cardiac output, Transfusion, Trauma

## Abstract

**Background:**

Intensive care of severe trauma patients focuses on the treatment of haemorrhagic shock. Tissues should be perfused sufficiently with blood and with sufficient oxygen content to ensure adequate tissue oxygen delivery. Tissue metabolism can be monitored by microdialysis, and the lactate/pyruvate ratio (LPR) may be used as a tissue ischemia marker. The aim of this study was to determine the adequate cardiac output and haemoglobin levels that avoid tissue ischemia.

**Methods:**

Adult patients with serious traumatic haemorrhagic shock were enrolled in this prospective observational study. The primary observed parameters included haemoglobin, cardiac output, central venous saturation, arterial lactate and the tissue lactate/pyruvate ratio.

**Results:**

Forty-eight patients were analysed. The average age of the patients was 39.8 ± 16.7, and the average ISS was 43.4 ± 12.2. Hb < 70 g/l was associated with pathologic arterial lactate, ScvO_2_ and LPR. Tissue ischemia (i.e., LPR over 25) developed when CI ≤ 3.2 l/min/m^2^ and Hb between 70 and 90 g/l were observed. Severe tissue ischemia events were recorded when the Hb dropped below 70 g/l and CI was 3.2-4.8 l/min/m^2^. CI ≥ 4.8 l/min/m^2^ was not found to be connected with tissue ischemia, even when Hb ≤ 70 g/l.

**Conclusion:**

LPR could be a useful marker to manage traumatic haemorrhagic shock therapies. In initial traumatic haemorrhagic shock treatments, it may be better to maintain CI ≥ 3.2 l/min/m^2^ and Hb ≥ 70 g/l to avoid tissue ischemia. LPR could also be a useful transfusion trigger when it may demonstrate ischemia onset due to low local DO_2_ and early reveal low/no tissue perfusion.

## Background

Trauma is a serious cause of morbidity and mortality. In adults up to 40 years of age, polytrauma is the most frequent cause of death [1]. The overall mortality rate in severely injured patients is approximately 30% [1], and following haemorrhagic shock, a loss of more than 40% intravascular volume can result in irreversible shock and death if it is not treated effectively [2].

Intensive care focuses on haemorrhagic shock and trauma-induced coagulopathy treatments during initial care. Shock is primarily a microcirculatory disorder, in which the oxygen supplied to tissues does not meet their metabolic demand, causing tissue ischemia. Manifest and occult shock is the result of decreased oxygen delivery due to low cardiac output and/or low oxygen content to tissues. Tissue ischemia may then develop, potentially causing multi-organ failure [3]. The physiological response to traumatic distress is intended to ensure oxygen delivery to vital organs, such as the brain and heart, by the centralisation of circulation and hyperdynamic circulation (i.e., high cardiac output). Distressed tissues with inadequate oxygen and substrate delivery react by switching their metabolism to the anaerobic pathway; lactate production in tissue is also proportional to the amount of energy produced [4, 5]. To avoid tissue ischemia and microcirculatory dysfunction, shock treatments must be effective, quick and as aggressive as possible to provide sufficient oxygen to distressed tissues. The global oxygen delivery (DO_2_) is determined by the haemoglobin concentration (Hb) and its saturation (SaO_2_), the amount of dissolved oxygen in the plasma and the cardiac output (CO); this can be defined by DO_2_ = COx(HbxSaO_2_x1.34 + PaO_2_x0.003). Tissues should be perfused with sufficient blood and oxygen content to ensure adequate tissue oxygen delivery; however, tissue requirements could differ from the global DO_2_ value. Monitoring tissue conditions could provide information about shock resolutions more quickly and precisely than global parameters, which include arterial lactate, ScvO_2_, Hb and CO levels. Tissue ischemia is poorly assessed by commonly used perfusion markers, and more detailed monitoring could be beneficial.

Haemostatic resuscitation, which is the primary treatment for haemorrhagic shock, attempts to restore and sustain tissue perfusion using an appropriate amount of fluid, transfusion and vasopressor administration; it also emphasises effective clotting and not impairing bleeding by increasing inadequate arterial pressure [6, 7]. Resuscitation guidelines have been developing particularly in the field of transfusions and fluids. Metabolic monitoring could be beneficial for the evaluation of resuscitation efficiency. New markers of ischemia could also help in decision-making processes.

Tissue ischemia is one of the primary causes of multi-organ dysfunction due to shock, which increases mortality [3]. Tissue conditions are not usually observed by traditional monitoring techniques, which typically only observe global parameters. Monitoring of tissue metabolisms could provide more precise assessments for shock management. Tissue metabolisms could be monitored by microdialysis. The lactate/pyruvate ratio (LPR) is a tissue ischemia marker [8], and LPR ≥ 25 indicates the onset of anaerobic metabolism [9]. The observed population consisted of severe blunt trauma patients, between 18 and 60 years of age with ISS > 25 and a blood loss estimated over 1 litre, who were admitted to the Emergency Department (ED). Measured parameters were of LPR, haemoglobin, cardiac output, arterial lactate and ScvO_2_. The aims of this study were to determine the adequate cardiac output and amount of haemoglobin required to prevent tissue ischemia; the study aims to define the association between the lactate/pyruvate ratio (LPR) on the haemoglobin (Hb) and the cardiac output (CO) or cardiac index (CI). The authors also compare LPR with arterial lactate and ScvO_2_. Adequate Hb and CI levels to avoid tissue ischemia were determined as outcomes.

## Methods

Severe blunt trauma patients with ISS > 25 between 18 and 60 years of age were enrolled in this prospective observational study between 2010 and 2013. Minimum required amount of 40 patients were enrolled due to preliminary power analysis. All participants presented with serious traumatic haemorrhagic shock with an estimated blood loss exceeding 1 l and hypotension (MAP ≤ 60 mm Hg). They were admitted to the Ostrava University Hospital Level 1 Trauma Centre Emergency Department as an inclusion criterion. Exclusion criteria included paediatric patients, penetrating trauma, non-serious haemorrhagia (blood loss up to 1 litre), ISS ≤ 25, no hypotension (MAP > 60 mm Hg), different ICU (non study) admission, dead in ED and dead within 6 hours from admission to ED; undergoing surgery was not an exclusion criterion. Monitoring was initiated as soon as possible and no later than 6 hours from admission. Study patients underwent prehospital care, ensuring treatment and transport. Diagnostic and therapeutic interventions were followed at the trauma centre according to the best clinical practises.

The observed parameters included haemoglobin concentration (Hb, haemoglobin g/l), central venous saturation (ScvO_2_, %), arterial lactate (L, mmol/l), cardiac output as cardiac index (CI, cardiac index, l/min/m^2^), and tissue lactate and pyruvate levels, which are displayed as the lactate/pyruvate ratio (LPR). The authors also recorded age, ISS, gender, weight, height, pre-hospital care time, and care time in the ED, theatre (if surgery was performed), and ICU. Haemoglobin, central venous saturation and arterial lactate were measured immediately after ED admission using a biochemical analyser (Roche Cobas b221 OMNI S) at 8-hour intervals but also at least three times for two hours following the administration of blood products. Cardiac output (CO) analysis was performed with a haemodynamic monitor (LiDCO Rapid), which analysed pulse characteristics in the arteria radialis beat-to-beat. Hourly CO averages were also recorded. Tissue monitoring was performed by extracellular fluid collected by a microdialysis probe inserted into certain tissues. Extracellular fluid samples were analysed in a biochemical bedside analyser. Tissue monitoring was performed using a CMA 60 microdialysis probe (CMA Microdialysis AB, Stockholm, Sweden), which was placed into each patient’s deltoid muscle. The authors used CMA Perfusion Fluid T1 dialysis solution (i.e., a lactate-free Ringer solution), and perfusion was accomplished with a CMA 106 pump at a constant flow of 0.3 μL/min. Subsequent analyses were performed with a CMA Iscus Flex analyser (CMA Microdialysis AB) using a set of reagents for the analysis of the lactate, pyruvate, glycerol and glucose levels (CMA Reagent Set A). These tissue values were analysed at 1-hour intervals. The data analysed included only those from the first 24 hours after trauma because shock is always eliminated after this period. All Hb, CI, ScvO_2_ and L levels were assigned to the corresponding LPR for the same moment in time for all patients. Then, data from all patients were divided into groups, which were created according to Hb and CI values: Hb ≤ 70 g/l; Hb 70-90 g/l; Hb ≥ 90 g/l and CI ≤ 3.2 l/min/m^2^ (i.e., “low”); CI 3.2-4.8 l/min/m^2^ (i.e., “normal”); and CI ≥ 4.8 l/min/m^2^ (i.e., “supranormal”). The measured variables (i.e., LPR, ScvO_2_ and arterial lactate) were also assigned to the created groups.

The study was performed in a single centre at the University Hospital in Ostrava. The Ethics Committee of the University Hospital Ostrava in the Czech Republic approved the study, which conformed to the tenets of the Declaration of Helsinki. Each of the awake and conscious study subjects signed the Informed Consent Form approved by the Ethics Committee of the University Hospital Ostrava. The Ethics committee waived the need to sign the Informed Consent in unconscious study subjects, who were unable to sign it.

**Figure 1:**
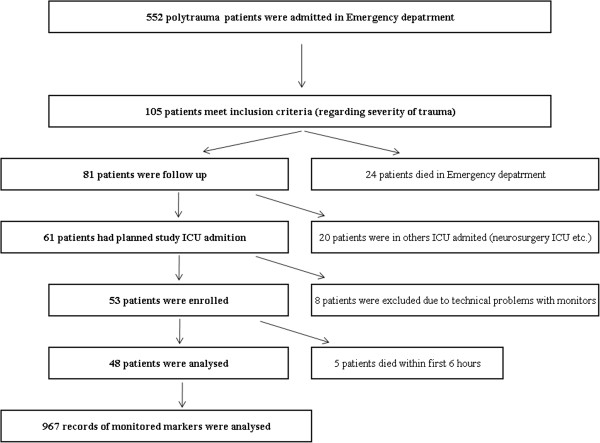
**Flow diagram of the study.**

The authors used R software (version 2.15.2) to perform the statistical analyses in this study. Missing values were extrapolated using a linear approximation with respect to measurement's date and time. The resulting p-values were adjusted for multiple comparisons using the Kruskall-Wallis test. Figure 1.

## Results

**Figure 2:**
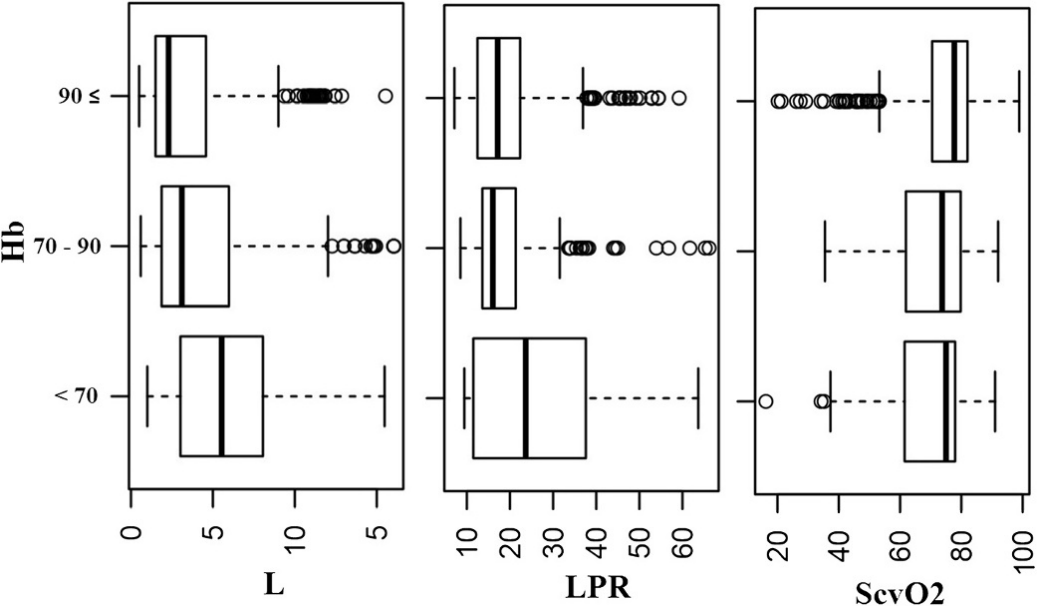
**Association of L, LPR and ScvO**_**2**_ **to Hb groups.** Hb, hemoglobin (g/l); L, arterial lactate (mmol/l); ScvO_2,_ central venous oxygen saturation(%); LPR, lactate/pyruvate ratio.

The authors observed an association between LPR on haemoglobin, cardiac output, ScvO_2_ and arterial lactate levels in the 48 patients. The authors analysed 967 records of LPR and the appropriate markers. The median time of pre-hospital care was 58 minutes; in the ED, patients spent 90 minutes, and the median time in theatre was 140 minutes. The average and median ages were 39.8 ± 16.7 and 36 years, respectively; the average and median ISS were 43.4 ± 12.2 and 43; and the average Hb was 97.70 ± 18.67 g/l Tables 1, 2 and Figure 2.

**Table 1:** **Demographics description of study population**

	Median	Percent
Age (years)	36	
ISS	43	
Male gender		83
Weight (kg)	82	
Height (cm)	178	
Pre-hospital care (minute)	58	
ED care (minute)	90	
Theatretime (minute)	140	
ICU stay (days)	10.5

**Table 2:** **Association of L, ScvO**_**2**_ **and LPR to Hb groups**

Hb groups	L		ScvO_2_		LPR	
g/l	mmol/l		Percent		Dimensionless	
	median	IQR	median	IQR	median	IQR
<70	5.538	5.036	65.00	16.47	28.66	26.08
70 - 90	3.105	4.095	73.7	17.72	16.02	7.68
>90	2.3	3.072	77.7	11.52	17.16	9.83

Hb, hemoglobin (g/l); L, arterial lactate (mmol/l); ScvO_2,_ central venous oxygen saturation(%); LPR, lactate/pyruvate ratio. Values are median ± interquartile range (IQR) (p < 0.05).

**Figure 3:**
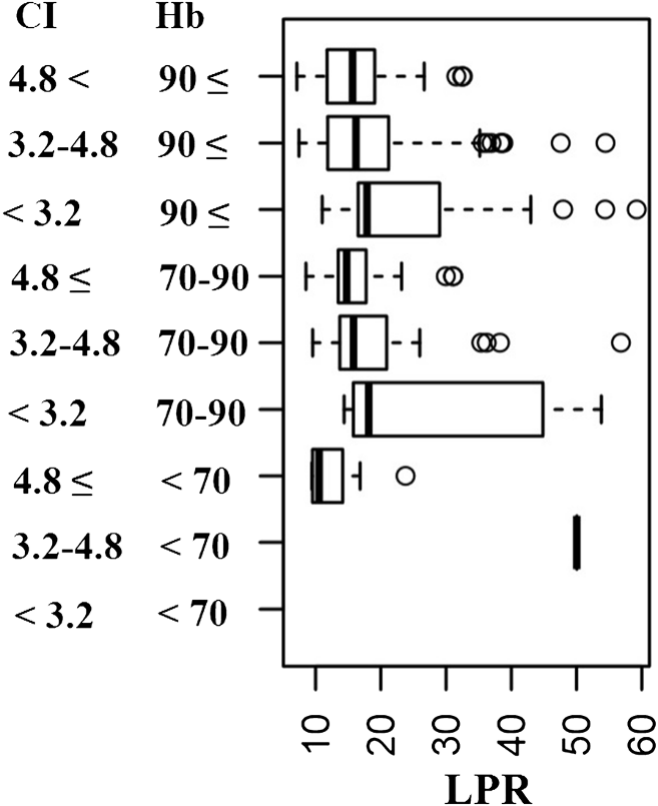
**Association of LPR (axis X) on Hb and CI groups simultaneously (axis Y).** CI, cardiac index (l/min/m^2^); Hb, hemoglobin (g/l); LPR, lactate/pyruvate ratio.

Monitored values during the first 24 hours after trauma are displayed in Table 2 and Figure 2. The median L, ScvO_2_ and LPR values and their association with Hb groups are also shown. Hb < 70 g/l was associated with pathologic arterial lactate, ScvO_2_ and LPR values. Pathologic arterial lactate in all Hb groups in the first 24 hours after trauma was observed, but higher Hb was associated with lower L. When Hb 70-90 g/l and Hb ≥ 90 g/l, ScvO_2_ and LPR was found to be normal Table 3 and Figure 3.

**Table 3:** **Association of lactate/pyruvate ratio (LPR) on hemoglobin (Hb) and cardiac index (CI) groups simultaneously**

	Hb groups					
CI groups	g/l					
l/min/m^2^	<70		70 - 90		>90	
	Median	IQR	Median	IQR	Median	IQR
<3.2			29.13	22.05	17.88	12.28
3.2-4.8	50.04	1.02	15.81	7.18	16.19	9.03
>4.8	10.54	3.29	14.76	4.24	15.67	7.27

CI, cardiac index (l/min/m^2^); Hb, hemoglobin (g/l); LPR, lactate/pyruvate ratio. Values are median ± interquartile range (IQR) (p < 0.05).

**Figure 4:**
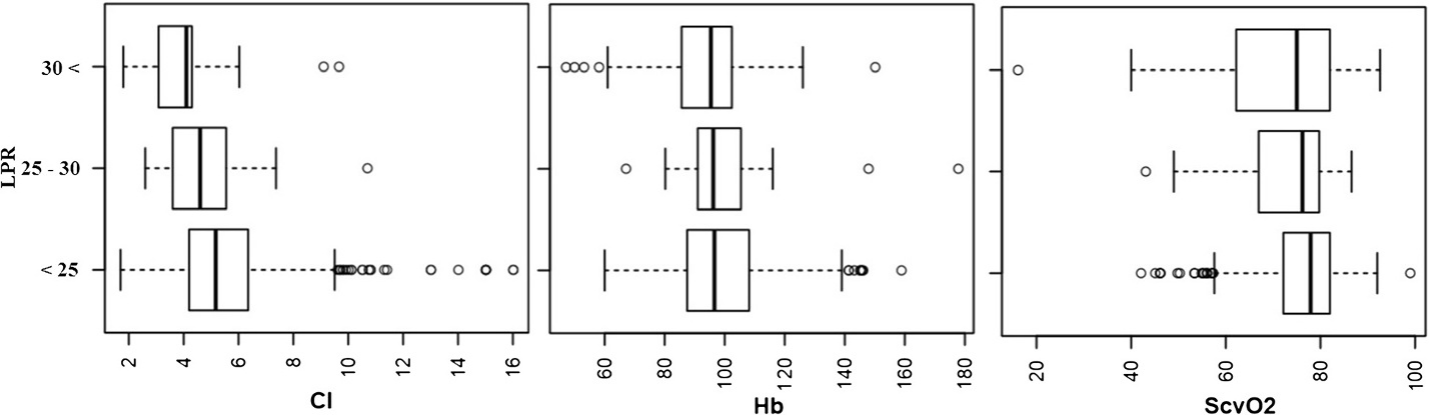
**Association of CI, Hb and ScvO**_**2**_ **on LPR devided to non-ischemic (<25), border ischemic (25-30) and severe ischemic (30<).** CI, cardiac index (l/min/m^2^); Hb, hemoglobin (g/l);); ScvO_2,_ central venous oxygen saturation(%); LPR, lactate/pyruvate ratio.

According to the concurrent values of Hb and CI, values of LPR are shown in Table 3 and Figure 3. Tissue ischemia developed when a low CI and haemoglobin between 70 and 90 g/l were observed. Severe tissue ischemia was registered when haemoglobin levels dropped below 70 g/l with a normal CI; CI above 4.8 l/min/m^2^ was not associated with tissue ischemia, even at haemoglobin values below 70 g/l. Note that CI below 3.2 l/min/m^2^ with concomitant haemoglobin values below 70 g/l were observed only in a few measurements; the statistical significance of LPR in these cases could thus not be determined. Another view of the data is shown in Figure 4, which displays cardiac output, haemoglobin and ScvO_2_ in association to LPR category (i.e., non-ischemic, border and severe ischemic LPRs). All results were statistically significant (p < 0.05) Figure 4.

## Discussion

Severe trauma patient transfusion management is challenging; the identification of both occult and inadequately resuscitated shock is a major clinical problem with traditional markers. Additionally, occult shock can present with normal global haemodynamics [10]. Severe polytrauma patient triage is crucial for good decision making regarding massive transfusion protocol (MTP) activation, which improves trauma haemorrhage outcomes [11]. Transfusion recommendations in trauma management attempt to maintain haemoglobin levels at 70-90 g/l [7]. Transfusions can be life saving but may also cause serious complications (i.e., TRALI, DIC, etc.) [12, 13]; inadequate transfusion and over-transfusion (Hb ≥ 110 g/l) are also harmful [14]. Fewer transfusions can lead to low DO_2_ with global or local (i.e., different local DO_2_ in tissues and organs according to local perfusion) ischemia development with regard to oxygen consumption. Many articles have discussed the benefits of the restriction transfusion strategy compared with liberal transfusions [14]. Haemoglobin could not be evaluated separately in traumatic haemorrhagic shock treatments; however, other markers may account for patient comorbidities (i.e., coronary disease), age and clinical status. Hb targets could differ according to the treatment period; Hb values of approximately 70 g/l could be low during initial treatments, particularly with a low CI or with severe comorbidities. This value could be sufficient in the next period of critical care, which starts after the initial shock and bleeding are resolved. Higher haemoglobin levels in the initial treatment period could reduce haemodilutions and decrease any potential ischemia if re-bleeding occurs; thus, mortality could be reduced [15]. Additional transfusion benefits could include restored blood viscosity and enhanced rheological properties of the blood. Transfusion of PRBC, which are the most frequent transfusion media, compared to fresh blood transfusions, may not provide immediate increase of oxygen delivery to tissues primarily due to transfusion storage length. A decrease in LPR could delay an increase in haemoglobin levels for 7-10 hours, and tissue ischemia could be eliminated over long time intervals [16]. Similarly, this phenomenon could be observed with LPR and ScvO_2_ trends, whereby a decrease in LPR could delay an increase in ScvO_2_ for 10 hours [16]. The average observed haemoglobin in the first 24 hours of this study, which included the suspected haemoconcentration period, were 97.70 ± 18.67 g/l; therefore, it could be speculated that if this value was approximately 70 g/l, then tissue ischemia could be eliminated at a much later period, and organ dysfunction could be worsened.

Additionally, fluid administration, which is a core treatment for shock and hypovolemia for preserving effective haemodynamic functions and tissue perfusion, has limits and adverse effects. Excessive fluid amounts lead to diluted coagulation factors, hypothermia [17] and endothelial glycocalyx damage [18]. Hypervolemia and fluid overload also lead to interstitium expansion. These influence the transcapillary gas and substrate exchange and decrease oxygen transport to tissues [19]. Hypovolemia leads to vasoconstriction and microvasculatory blood flow restriction, which cause ischemia due to low oxygen and substrate delivery [19]. Tissue perfusion could be altered with the administration of vasoactive agents, and knowledge of metabolic tissue conditions (i.e., LPR) during haemostatic resuscitation could help guide treatment.

Lactataemia evaluation in shock is typically difficult in practice; elevated arterial lactate levels are associated with increased mortality and morbidity [7], and lactate normalisation is one of most frequent resuscitation targets. Arterial lactate could be elevated without clinical signs of shock but could also be an indication of on-going ischemia [20]. In contrast, during a shock state, lactate could accumulate in low or non-perfused tissues, and serum levels could be falsely determined to be low. After tissue perfusion is restored, lactate levels increase as a sign of reperfusion, and high lactate levels could be connected with normal aerobic metabolism. The normalisation of lactate values depends on the hepatic clearance of lactate or its consumption in tissues, which could also decrease during shock. It is expected that lactate levels will follow the oxygen debt, but in some clinical conditions, lactate levels may normalise without the resolution of tissue oxygen debt [21]. There are also a number of non-ischemic factors that elevate lactate levels (e.g., stress and catecholamines) [22]. Arterial lactate levels should, therefore, be critically evaluated because assessment of the lactate levels alone fails to discriminate between ischemia and aerobiosis [23].

The measurement of LPR could be more useful than that of arterial lactate levels alone when discriminating between occult shock with ischemic tissue conditions and aerobic metabolism. LPR may be used as an early indicator of emerging ischemia during shock [24, 25] and could also help to discriminate between elevated ischemic or non-ischemic lactate levels and to distinguish between the anaerobic aspects of hyperlactataemia [26]. Hyperlactataemia with elevated LPR levels is associated with higher mortality than hyperlactataemia with normal LPR levels [26]. LPR levels over 25 indicate anaerobic metabolism onset [9] and are a more precise marker of ischemia than lactate levels alone [27].

ScvO_2_ is a frequently used marker that is useful in guiding fluid, catecholamine and transfusion therapy [28]. It is a global parameter of the oxygen extraction sum from the blood, and therefore, normal values do not exclude severe local tissue damage or regional tissue ischemia. It is expected that low ScvO_2_ could reflect low DO_2_. The most respected target value for shock resuscitation is an ScvO_2_ value above 70% [29]; however, emerging ischemia originates at the cellular level, and changes in the LPR value could precede that in the ScvO_2_ value by 10 or 11 hours [16]. A low ScvO_2_ could lead to DO_2_ manipulation, but a normal or high ScvO_2_ could lead to false satisfaction due to treatment, allowing background cell dysfunction to occur.

Haemodynamic monitoring of CO is typically the next most frequent measurement in the evaluation of traumatic haemorrhagic shock. Trauma patients may have a low CI in the first hours after trauma (i.e., the ebb phase of shock). Tachycardia could then occur to preserve CI during hypovolemia, and later, CI rises to supranormal values to act as a physiologic reserve marker and as a compensatory reaction to overcoming distress (i.e., the flow phase of shock). In contrast, a normal CI value may not ensure adequate tissue perfusion [10]. Many authors have examined the evaluation of the VO_2_/DO_2_ relationship. A normalised VO_2_ seems to be essential for organism recovery, and oxygen supply independency is a key strategy for haemodynamic optimisation [30]. Increasing DO_2_ to supranormal values, however, was found to be beneficial in some studies [31], but not in others [32]. Additionally, excessive oxygen supply may be deleterious due to ineffective metabolic costs; however, it may be reasonable to increase the DO_2_ to 20% above the critical DO_2_ value (i.e., the limit of DO_2_/VO_2_ dependency) [30]. Velmahos GC demonstrated that patients who achieved supranormal haemodynamic parameters (i.e., CI > 4.5 l/min/m^2^, DO_2_I > 600 ml/min/m^2^ and VO_2_I > 170 ml/min/m^2^) after severe trauma had better outcomes than patients who did not achieve those limits [31]. A spontaneously high DO_2_ could be used as a simple physiologic reserve marker and a predictor of outcomes. An excessive artificial increase in DO_2_ without a functional organism reserve could be detrimental [31]. Resuscitation efforts should be limited to what is only necessary with respect to human variability [33].

In this study, Hb < 70 g/l (i.e., without a CI distinction, whereby different CI are included) was associated with pathologic lactate, ScvO_2_ and LPR values; therefore, treatment interventions or more intensive monitoring were inevitable (Table 2.). Pathologic blood lactate levels in all Hb intervals were also observed and certainly influenced the reduced lactate clearance during the first 24 hours after trauma.

To eliminate tissue ischemia (i.e., normalise LPR), it is rational to increase the DO_2_, but only in patients with Hb values of 70-90 g/l and a low CI; with a normal CI and Hb < 70 g/l; or, most likely, with a low CI and Hb < 70 g/l (Table 3.). An artificial CI increase to supranormal values could be beneficial, but only if it is accomplished when Hb < 70 g/l to avoid an ischemic LPR; however, potential adverse vasoactive medication effects may occur. It may be beneficial to transfuse to Hb 70-90 g/l and a normal CI, which will also lead to a normal LPR. An ischemic LPR with supranormal CI values was not observed, supporting the benefits of the physiologic reaction to trauma discussed above. Increasing a low CI to normal is rational in patients with Hb levels at 70-90 g/l and most likely <70 g/l. In cases where Hb > 90 g/l, the increase of a low CI to normal levels leads to a decrease in LPR; however, the LPR in both of these situations is under the ischemic threshold. A low CI should be avoided.

To avoid tissue ischemia, transfusions are applicable in patients with Hb < 70 g/l and a normal CI and most likely in low CI cases as well. An Hb increase to over 90 g/l could only be warranted when patients have a low CI; however, it is preferable to increase a low CI. With regard to LPR, it was shown that supranormal CI levels could be used to treat tissue ischemia, but the artificial elevation of CI could be dangerous [31]. Another requirement is to adequately correct the blood oxygen content using not only Hb but also SaO_2_ and PaO_2_.

### Limitations

This study has several limitations. Tissue monitoring in the early phase of management of severe trauma patients is typically difficult. In this timeframe, physicians employ several necessary interventions, including diagnostic and therapeutic methods. The observations of this study were performed in addition to and did not influence the standard care of the examined critically ill patients; tissue monitoring was begun within 6 hours after admission. The study was performed in full working Trauma centre. The authors enrolled only 48 patients, and each patient was enrolled by a physician associated with this study. The authors also had limited human and economic resources. The study was financial supported partially by a grant, and certain technical problems with encountered with equipment during testing.

## Conclusion

LPR is shown to be a useful marker to manage traumatic haemorrhagic shock therapies. LPR is the result of cell metabolic functions and thus reflects the sum of all interventions. LPR target therapies could be better than traditional management, which use other global markers that only assume good tissue conditions; tissue conditions could be directly monitored by the proposed method. In initial traumatic haemorrhagic shock treatments, it may be better to maintain CI ≥ 3.2 l/min/m^2^ and Hb ≥ 70 g/l. Additionally, LPR may be very important and useful as a transfusion trigger in normal or low CI situations with decreased haemoglobin; it could be used to predict the onset of ischemia due to low local DO_2_ levels and effectively reveal low tissue perfusion.

## Abbreviations

CO: Cardiac output
PaO_2_: Partial pressure of oxygen in arterial blood
Hb: Haemoglobin
LPR: Lactate/pyruvate ratio
CI: Cardiac index
SaO_2_: Arterial saturation of oxygen
PRBC: Packed red blood cells
MTP: Massive transfusion protocol
TRALI: Transfusion-related acute lung injury
ScvO_2_: Central venous oxygen saturation
VO_2_: Oxygen consumption
DO_2_: Oxygen delivery
VO_2I_: Oxygen consumption index
DO_2I_: Oxygen delivery index
L: Arterial lactate
ISS: Injury severity score
ED: Emergency department
DIC: Disseminated intravascular coagulation.
